# Evaluation of a Bladder Cancer Cluster in a Population of Criminal Investigators with the Bureau of Alcohol, Tobacco, Firearms and Explosives—Part 1: The Cancer Incidence

**DOI:** 10.1155/2012/101850

**Published:** 2012-12-09

**Authors:** Susan R. Davis, Xuguang Tao, Edward J. Bernacki, Amy S. Alfriend

**Affiliations:** ^1^Federal Occupational Health, Department of Health and Human Services, 4550 Montgomery Avenue, Suite 950, Bethesda, MD 20814, USA; ^2^Division of Occupational and Environmental Medicine, Department of Medicine, Johns Hopkins University School of Medicine, 615 N. Wolfe Street, Baltimore, MD 21205, USA

## Abstract

This study investigated a bladder cancer cluster in a cohort of employees, predominately criminal investigators, participating in a medical surveillance program with the United States Bureau of Alcohol, Tobacco, Firearms and Explosives (ATF) between 1995 and 2007. Standardized incidence ratios (SIRs) were used to compare cancer incidences in the ATF population and the US reference population. Seven cases of bladder cancer (five cases verified by pathology report at time of analysis) were identified among a total employee population of 3,768 individuals. All cases were white males and criminal investigators. Six of seven cases were in the 30 to 49 age range at the time of diagnosis. The SIRs for white male criminal investigators undergoing examinations were 7.63 (95% confidence interval = 3.70–15.75) for reported cases and 5.45 (2.33–12.76) for verified cases. White male criminal investigators in the ATF population are at statistically significant increased risk for bladder cancer.

## 1. Introduction

The Bureau of Alcohol, Tobacco, Firearms and Explosives (ATF), a law enforcement agency within the United States Department of Justice, has been in the business of investigating the cause and origin of fires and explosions since the 1970s. ATF typically collaborates with other federal, state, and local authorities to provide this service through several venues. At the local level, criminal investigators with ATF, especially ones holding special designations as certified fire investigators (CFIs), work side-by-side with their state and local counterparts to assist with investigation of post-fire and post-blast scenes. These individuals may also serve on city-based federal arson task forces, in partnership with other federal, state, and local investigators, to address acute arson problems. At the national level, ATF maintains a well-trained national response team (NRT) which provides comprehensive response to assist these other jurisdictions with onsite investigation of major arson and bombing incidents. The NRT is a multispecialty team comprised of criminal investigators with post-fire and post-blast investigation expertise, explosives enforcement specialists, forensic chemists, fire protection engineers, and other technical experts. ATF established the NRT program in 1978 and subsequently instituted a program for certification of criminal investigators as fire investigators (CFIs) in 1986. 

 In 1995, ATF commenced a voluntary medical surveillance program for members of the NRT to monitor the health of participants working in the potentially hazardous environment of the post-fire/post-blast scene. The agency extended the voluntary program to all CFIs in 1997. By 2000, three white male participants of the program had reported diagnoses of bladder cancer between the years 1994 and 1999. All three individuals were nonsmokers and younger than 50 years of age when diagnosed. This information raised concern that a bladder cancer cluster was occurring among scene investigators. In response to this concern, in 2002 ATF mandated participation in the medical surveillance program for all NRT members, all explosives enforcement specialists, and all criminal investigators, including those not involved in post-fire/post-blast investigations. To facilitate future epidemiologic evaluation of the significance of the apparent cancer cluster, ATF also committed medical surveillance data entry into an electronic database. By 2006, four additional white male program participants had reported diagnoses of bladder cancer between 2001 and 2005. Combined, the seven reported cases ranged in age from 32 to 53 in the year of diagnosis. From a job title perspective, all cases were among criminal investigators, who comprised 96% of the employees participating in the surveillance program. In addition, six of the seven cases reported working post-fire and post-blast scenes while employed with ATF. None of the seven cases reported work histories which involved investigation of such scenes prior to their employment with ATF. 

The presence of this cancer cluster generated two questions. First, was this group of employees, predominantly criminal investigators, experiencing a greater than expected incidence of bladder cancer from a demographic perspective? Second, was post-fire/post-blast scene investigation associated with increased risk for bladder cancer? Part 1 of this study addresses the question on cancer incidence and Part 2 addresses the question on cancer risk associated with investigation of these scenes. Analyses for both parts use data collected through the medical surveillance program. 

In the United States, bladder cancer is expected to be the sixth most commonly occurring cancer, excluding basal and squamous cell skin cancers, in 2012 [1]. As reported by the American Cancer Society, the estimated number of new bladder cancer cases in 2012 is 73,510, which equates to approximately 4.5% of all new cases of cancer [1]. The demographic characteristics associated with greatest risk for bladder cancer include male gender, white race, and increasing age [2]. According to the surveillance epidemiology and end results (SEER) age-adjusted incidence data for the period 2005–2009, bladder cancer occurs four times more often in men than in women [3]. In this data set, it ranks as the fourth most common cancer among men, but only the twelfth most common cancer among women [3]. The incidence among whites is 1.78 times greater than the incidence among blacks, while the incidence among Hispanics is 0.90 times the incidence among blacks [2, 3]. Bladder cancer incidence is the lowest among Asian/Pacific islanders and American Indian/Alaska natives [3]. The risk of onset increases with age over the age of 40 and is greatest during the ninth decade of life [3]. About 90% of cases occur in individuals over the age of 55, with the average age being 73 at the time of diagnosis [2]. In the ATF cancer cluster, all the cases of bladder cancer represented both the gender and the race with the greatest incidence of bladder cancer, but the ages of the cases at the time of diagnosis were relatively young for bladder cancer as all cases occurred in individuals less than 55 years of age. 

Recognized nondemographic risk factors include cigarette smoking, bladder birth defects, chronic bladder inflammation, genetic predisposition, use of herbal remedies containing aristolochic acid, drinking water containing arsenic and chlorination by-products, prior history of bladder cancer, chemotherapy and radiation therapy, and specific industries and occupations with exposures to known or suspect bladder carcinogens [2, 4–13]. Many studies have explored the association of fluid intake and type of fluid intake with risk for bladder cancer, but findings for both total fluids and specific fluids have been inconsistent, likely due to influences of confounding variables such as the presence of bladder carcinogens in tap water [14–24]. Two studies have demonstrated that increased frequency of urination is associated with reduced risk for bladder cancer [18, 21]. 

Smoking, the number one risk factor for bladder cancer, is estimated to cause approximately 50% of bladder cancer cases in men and 30% of cases in women [6]. Second to smoking, occupational exposures to carcinogens may account for as few as 10% of cases in men and five percent of cases in women to as many as 20–25% of all bladder cancers [6, 11]. Established at risk industries include the manufacturing of products such as synthetic dyes and paints, cables, textiles, leather works, and aluminum and the petrochemical, coal tar, and rubber industries [5–7, 12, 25–30]. A number of specific occupations have also been identified to be associated with increased risk of bladder cancer. These include, but are not limited to, cooks and kitchen workers, electricians, hairdressers, leather workers, machinists, petroleum workers, rubber workers, coal miners, truckers, and vehicle mechanics, as summarized by Schulte et al. [12] in 1987, as well as coke oven workers, roofers, dry cleaners, chimney sweeps, and painters, as addressed by others in more recent literature [5–7, 25, 31–34]. Exposure to both cigarette smoke and occupationally related bladder carcinogens may work synergistically to increase further the risk for bladder cancer [2, 7]. 

As an occupation, the broad job category of law enforcement is generally not recognized for being at increased risk for bladder cancer. In 1987, Schulte et al. [12] did include protection guards on their summary list of occupations associated with risk for bladder cancer, but acknowledged that the potential etiologic agent was unknown. As the rate of occurrence of bladder cancer is almost five times greater than the associated death rate in the US, with 80% of cases surviving for five or more years after diagnosis [1], cancer incidence is a more sensitive measure of occupational risk for bladder cancer than related mortality and it is important to differentiate between epidemiologic studies looking at cancer incidence and those looking at cancer mortality. Many epidemiologic studies on the association of bladder cancer incidence and occupations have not found police officers, guards, and related categories, such as protective services and government worker inspection/investigation occupations, to be associated with increased risk for bladder cancer [11, 35–46]. One exception was a study by Howe et al. [47] which showed guards and watchmen to have a statistically significant age-adjusted relative risk for bladder cancer of 4.0. In Reulen et al.'s [32] 2009 meta-analysis on the association between bladder cancer incidence and occupation, summary relative risks for bladder cancer were obtained for protective service occupations from the findings of 23 studies and for police officers and guards from the findings of 14 studies. These summary relative risks were only marginally elevated and not statistically significant, at 1.07 (95% confidence interval (CI) 0.96–1.19) for protective services and 1.10 (95% CI 0.95–1.29) for police officers and guards. Three mortality studies on police officer cohorts also did not find statistically significant increases in mortality from bladder cancer overall [48–50]. One of these studies did demonstrate that policemen who were professional drivers had significantly increased bladder cancer-related mortality [48] and another showed a higher than expected mortality rate for police officers with 10–19 years of service [50]. As some, but not all, epidemiology studies have demonstrated an increased risk for bladder cancer in jobs with exposure to diesel or traffic fumes [6, 40, 43, 51, 52] and one meta-analysis has shown increased cancer risk for several types of vehicle drivers [32], the use of broad law enforcement job categories in studies on cancer risk and occupation assumes a degree of uniformity in exposures to potential bladder carcinogens and risk for bladder cancer, which may not always be the case. 

Part 1 of this study investigates the statistical significance of the incidence of bladder cancer in the ATF employee population comprised of criminal investigators and members of the NRT.

## 2. Methods 

### 2.1. Study Time Frame and Study Subjects

The time interval of this study is 1993, the year preceding diagnosis of the first bladder cancer case, through 2007. For this period, a full roster cohort was constructed from the annual staffing rosters provided by ATF for each calendar year from 1993 through 2007. The annual staffing rosters included all criminal investigators, explosives enforcement specialists, forensic chemists, fire protection engineers, and a small number of other specialists typically affiliated with NRT work, regardless of individual membership on the NRT or eligibility for participation in the medical surveillance program, who were currently working for ATF in each respective calendar year. ATF provided the demographic study parameters of gender, race, and age, and the job series and titles for all members of the full roster cohort. Members of the full roster cohort are dispersed throughout the United States and its territories Puerto Rico and Guam and may move around within this geographic area during their employment with ATF. 

With the advent of the medical surveillance program in 1995, a subset of the full roster cohort, comprised of employees participating in the program, was created. As explained in the introduction, the program was initially voluntary and offered to members of the NRT, but became mandatory in 2002 for all criminal investigators, explosives enforcement specialists, and members of the NRT. Setup in partnership with Federal Occupational Health (FOH), United States Department of Health and Human Services, the program consisted of an annual evaluation which included a medical history and tobacco use questionnaire, physical examination, and laboratory and ancillary tests. Collection of detailed work history information, including job series and titles, began in 2003 with the institution of a work history questionnaire. The use of FOH's electronic database, the Occupational Health Information Management System (OHIMS), to facilitate future epidemiologic evaluation of the bladder cancer cluster began in 2002. Data for key study variables from pre-2002 exams were retrospectively retrieved and also entered into OHIMS. 

For the cohort of employees participating in the medical surveillance program from 1995 to 2007, pertinent data collected with each annual evaluation included the demographic variables, gender, race, and age, as well as cancer history and tobacco use history. Data on job series and titles were also provided by members of the cohort participating in the program between 2003 and 2007. The demographic data and the job series and titles data provided by ATF for the full roster cohort were subsequently cross-referenced with the same data provided by employees participating in the medical surveillance program. Any data inconsistencies between the two sources were resolved by medical record review or phone contact with employees. 

### 2.2. Identification and Verification of Bladder Cancer Cases

As stated in the introduction, all bladder cancer cases were initially identified by employees self-reporting the diagnosis and year of diagnosis at the time of the annual medical surveillance evaluation. The self-reported cases were subsequently contacted by the occupational medicine physician overseeing the medical surveillance program, who informed them of the bladder cancer cluster and of the plans to evaluate the significance of the cluster through epidemiologic analysis and requested their assistance with voluntary provision of a pathology report for verification of case diagnosis. Requests for pathology reports were accompanied by provision of medical release forms for completion by cases. 

### 2.3. Study Design

This is a cohort study in which standardized incidence ratios (SIRs) were calculated for four defined populations of ATF employees to compare the observed bladder cancer case numbers in each ATF population with the expected cancer case numbers based on incidence rate in the US general population, appropriately adjusted for age and stratified by sex and race, as relevant to each of the four ATF populations. The US general population was chosen as the best reference population for analysis due to the nation-wide dispersion of ATF employees under study. As all the bladder cancer cases occurred among white male criminal investigators, the populations chosen for analysis included: (1) the full roster cohort comprising all males and females, (2) all white males in the full roster cohort, (3) all white males in the full roster cohort with examinations (cancer and tobacco use histories), and (4) all white males in the full roster cohort with both examinations and work histories who were also criminal investigators. Determination of the expected cancer incidence rate was based on data from the surveillance epidemiology and end results (SEER) program for the US population for the period 1993–2007. The SIR estimates the relative risk of bladder cancer incidence in the ATF population compared to the US population adjusted for age and stratified by race and gender. Computations for each population included determination of the person-year distribution during the study period, which served as the denominator for the respective SIR analysis. One person-year was counted for each year an individual was a member of the cohort. The person-years were arranged by five-year age increments and three five-year time intervals, 1993–1997, 1998–2002, and 2003–2007. 

## 3. Results 

### 3.1. Study Subjects 

Table 1 shows the distribution of individuals by gender and race for the full roster cohort, for the subset of employees with surveillance examinations, for the subset of employees with surveillance examinations and work histories, and for the subset of criminal investigators with surveillance exams and work histories. The percent distribution of individuals by gender and race for each of four of these populations is comparable. 

The full roster cohort comprised 3,768 individuals (Table 1). Criminal investigators, with job series 1811, accounted for 96% of all employees in the full roster cohort and 96% of all white males in the full roster cohort. Within the full roster cohort, only 18.2% of members were non-white and only 12.6% of members were female. 

Of the full roster cohort, 2,712 (72%) members participated in the medical surveillance program between 1995 and 2007 and had data for one or more examinations in the program's electronic database (Table 1). The percentage of full roster cohort members with examinations was not greater than 72% due in part to the medical surveillance program being voluntary and only open to members of the NRT and to CFIs prior to 2002. Since 2002, when the program became mandatory for all criminal investigators, explosives enforcement specialists, and members of the NRT, the percentage of currently employed cohort members who obtained annual examinations ranged from a low of 50% in 2003 to a high of 67% in 2006. Despite this variance in annual rate of participation in the mandatory program, 2697 of 3136 (86%) full roster cohort members who were employed for one or more years between 2002 and 2007 obtained at least one medical exam. 

Job history data, collected from employees between 2003 and 2007 at the time of the annual exam, were available for 2549 (68%) members of the full roster cohort. Of these individuals, 2478 (97%) were criminal investigators (Table 1).

### 3.2. Characteristics of Bladder Cancer Cases

During the study period, seven individuals reported bladder cancer diagnoses. At the time of the analysis, five of the seven cases had provided medical documentation (pathology reports) verifying the diagnosis of bladder cancer and diagnosis year. Another case provided verifying documentation following the completion of the analysis. 

 As affirmed from review of pathology reports of the urinary bladder biopsies of the five cases verified at the time of the analysis, four of the cases were low grade papillary transitional cell carcinomas and one was transitional cell carcinoma *in situ*. The subsequent sixth case verified after analysis was also low grade papillary transitional cell carcinoma.

The first case was diagnosed in 1994 and the most recent case was diagnosed in 2005. As already known from the medical surveillance program, all bladder cancers occurred in white males and in criminal investigators. Table 1 includes the distribution of reported bladder cancer cases by gender and race for each defined employee population. White males comprised 72% (2723/3768) of the full roster cohort and 69-70% of each of the three subset populations, including criminal investigators with medical surveillance examinations and work histories (69% (1715/2478)). The cases ranged from 32 to 53 years of age the year of diagnosis, with three individuals being in their 30s, three being in their 40s, and one being in his early 50s.  Table 2 shows the distribution of the self-reported bladder cancer cases at the time of diagnosis in the same five-year age increments and three five-year time intervals as used to establish the person-year distributions for each SIR analysis. Two cases were diagnosed in 1993–1997, four cases were diagnosed in 1998–2002, and one case was diagnosed in 2003–2007. 

### 3.3. Distribution of Person-Years within the Study Populations Undergoing Incidence Analysis

Table 3 summarizes the total person-years calculated for each of the four study populations undergoing incidence analysis. The number of total person-years ranged from a high of 34,818 for the full roster cohort to a low of 17,976 for the population of white male criminal investigators with exams and work histories. The pattern of distribution of person-years was similar for all four study populations and is illustrated in Table 2 for the population of white males in the full roster cohort. The vast majority of employees for each study population fell within 25 and 54 years of age for each of the five-year time intervals. This distribution pattern reflects ATF's practice of hiring criminal investigators with prior work experience and the mandated retirement age of 57 years for federal criminal investigators. 

### 3.4. Bladder Cancer Incidence Ratios

Table 3 presents the standardized incidence ratios (SIRs) calculated for each of the four defined study populations: the full roster cohort, all white males in the full roster cohort, all white males with examinations (cancer and tobacco use histories), and all white males with examinations and work histories who were also criminal investigators. To access the effect of the two unverified cancer cases on SIR outcomes, SIRs were performed for the scenario with seven reported cancer cases and for the scenario with five verified cases for each of the four study populations. 

When computed with all seven cases, the SIR is 2.41 (95% CI 1.17–4.96) for the entire roster cohort, 2.93 (95% CI 1.42–6.07) for the white male cohort, 6.08 (95% CI 2.94–12.54) for white males with exams, and 7.63 (95% CI 3.70–15.75) for white male criminal investigators with exams and work histories (Table 3). When recalculated with only the five verified cases, the SIR is 1.72 (95% CI 0.73–4.02) for the entire roster cohort, 2.09 (95% CI 0.90–1.91) for white males, 4.34 (95% CI 1.85–10.16) for white males with exams, and 5.45 (95% CI 2.33–12.76) for white male criminal investigators with exams and work histories (Table 3). The elevated SIRs are statistically significant for all four of these populations when all seven cases are included in the analysis and remain statistically significant for white males with exams and white male criminal investigators with exams and work histories when only the five verified cases are used. 

 Age-specific cancer incidence rates in the white male ATF population were greater than the rates for the adjusted U.S. reference SEER population for each age group in which cases occurred (Table 4). This finding is expected since 90% of bladder cancers occur in individuals over the age of 55 and all seven cases in the ATF population were younger than 55 years at the time of diagnosis. The highest age-specific relative risk for bladder cancer in the ATF population compared to the reference population, and the only one of statistical significance, was seen in the 30–34 age group, the youngest age group experiencing bladder cancer within the ATF population. 

## 4. Discussion

Recognition of a bladder cancer cluster among white male criminal investigators participating in an ATF medical surveillance program raised concern that the employee population was experiencing a greater-than-expected incidence of bladder cancer and that post-fire/post-blast scene investigation might be associated with increased risk of bladder cancer. Part 1 of this epidemiologic study determined that bladder cancer incidence in the white male study population was significantly elevated statistically for the period 1993–2007.

This study illustrates the twofold utility of using a medical surveillance program to monitor the health of an employee population potentially exposed to hazardous agents and to perform epidemiologic analysis of the significance of cancer occurring within the population. Although the ATF medical surveillance program evolved over time in several ways, which included changing from voluntary to mandatory participation, and despite an annual participation rate of 50–67% after the program became mandatory, the requisite data were sufficient to perform SIRs for the ATF study population which showed white males to be at increased risk for bladder cancer when compared to white males in the US reference population. 

All bladder cancer cases in the ATF cohort were reported by white males, who constituted about 72% of the full roster cohort. In the incidence analyses of the two larger ATF populations, the full roster cohort and white males in the full roster cohort, the cancer risk was elevated for analyses performed with both seven reported and five verified cases, but was only statistically significant for the analyses with the seven reported cases. Since these two ATF populations included individuals who had not participated in the medical surveillance program and whose cancer history was unknown, which amounted to 28% of the full roster cohort and 31% of white males in the full roster cohort, the actual number of cases of bladder cancer within these two populations could be greater than the observed seven reported and five verified cases, which would lead to even higher SIRs than the ones calculated with the seven and five cases. With the two smaller ATF populations, white males with exams and white male criminal investigators with exams, the computed SIRs were elevated and statistically significant with both seven reported and five verified cancer cases. The SIRs of the two smaller populations, understandably higher than those of the two larger populations, might best depict the true cancer risk for the ATF cohort as the reported cancer history is known for all individuals in these two smaller populations.

The finding that white male criminal investigators in the ATF population are at increased risk for bladder cancer is in contrast with the findings of prior epidemiologic studies of bladder cancer incidence in law enforcement occupations, in which law enforcement and related job categories were generally found not to be at increased risk for bladder cancer [11, 32, 35–39, 41, 42, 44–46]. In Reulen et al.'s [32] meta-analysis, the summary relative risk was 1.10 (95% CI 0.95–1.29) for police officers and 1.07 (95% CI 0.96–1.19) for protective service occupations. What may make this population of ATF criminal investigators unique from other populations of law enforcement specialists is the presence of a sizable subset of ATF criminal investigators who specialize in investigation of post-fire and post-blast scenes. What we know from the medical surveillance program is that six of the seven bladder cancer cases in the current study had occupational histories of working these scenes while employed with ATF. Thus, the increased incidence of bladder cancer identified among white male criminal investigators in the current study appears to be associated with the performance of post-fire/post-blast investigations. If this is the case, the increased risk for bladder cancer in this subset of specialized criminal investigators is sufficiently strong to influence the bladder cancer risk within the entire ATF study population. 

The magnitude of the SIRs computed in this study deserves some discussion. For the population of white male criminal investigators with exams, the SIRs were 7.63 (95% CI 3.70–15.75) for seven reported cases and 5.45 (95% CI 2.33–12.76) for five verified cases, and for the slightly larger population of all white males with exams, the SIRs were 6.08 (95% CI 2.94–12.54) for seven reported cases and 4.34 (95% CI 1.85–10.16) for five verified cases. In individual epidemiologic studies of bladder cancer incidence in other occupations and industries, statistically significant elevations in relative risk have been found with a 1.1-fold to fivefold increase [11, 32, 33, 39, 41–44, 46, 52–54] and even with a sixfold to tenfold increase for some occupations such as chemical workers [41, 47], dye manufacturing [55, 56], railroad workers [47], and physicians [41]. In one epidemiologic study on firefighters, the reported statistically significant odds ratio for bladder cancer was as high as 22.7 [41]. Thus, some epidemiologic studies on other occupations have obtained elevated relative risk for bladder cancer of the same order of magnitude as that found in the present study on criminal investigators. In Reulen et al.'s [32] meta-analysis on the association between bladder cancer and occupations, however, statistically significant summary relative risks which were found for several occupations only fell in the 1.1–1.3 range. These occupations included miners, rubber workers, leather workers, four types of professional drivers, and mechanics, but not chemical workers, firefighters, police officers, protective service occupations, or health care professionals. 

The elevated risk for bladder cancer in the current study cohort also approximates the increased risk for bladder cancer seen in smokers compared to nonsmokers, as demonstrated in various epidemiologic studies which show smokers to have a twofold to sixfold increased risk for bladder cancer [1, 6, 57–59]. Further corroborating study is advised to verify the magnitude of the increased risk found among criminal investigators in the current study.

Although the elevated SIRs for the two populations with exams remained statistically significant when analyzed with only the five verified cases, having two of the seven reported cases (28%) unverified at the time of statistical analysis constitutes a weakness of the study and illustrates a limitation of using data from a medical surveillance program to conduct a cancer incidence analysis. With so few total cases, the difference in number between reported and verified cases can impact the significance of study outcomes, as demonstrated by comparing the SIRs computed with both the seven reported and the five verified cases for the full roster cohort and for white males in the full roster cohort. Since the study analysis was completed, ongoing effort to verify the unverified cases was successful in verifying one of the two cases. 

Another point to make is that the SIRs on the populations with exams could in actuality be artificially high, as only 72% of individuals in the full roster cohort underwent physical examination and individuals without medical issues may have selectively avoided coming in for exams. Since the institution of the mandatory program in 2002, however, 86% of individuals employed by ATF between 2002 and 2007 obtained at least one exam during this time frame and these 2697 employees accounted for 99% of the 2712 individuals in the full roster cohort with exams. With this level of participation in the mandatory program since 2002, potential for bias due to exam avoidance is likely very limited.

 In conclusion, white male members of the ATF cohort experienced statistically significant increased risk for bladder cancer when compared to white males in the US population for the study period 1993–2007. Among white males with exams and white male criminal investigators with exams, the elevated risk was demonstrated for computations with both seven reported and five verified cancer cases. There was no observed bladder cancer risk for nonwhite males and all females in the cohort study. 

With six of the seven cases in the bladder cancer cluster, having known histories of investigating post-fire and post-blast scenes while employed with ATF, scene investigation work appeared to be linked with the observed increase in bladder cancer incidence. Part 2 of the study will evaluate the association of post-fire/post-blast scene work history and risk for bladder cancer, while controlling for tobacco use history. 

## Figures and Tables

**Table 1:** Distribution of self-reported bladder cancers and employees in key ATF study populations by gender and race (1993–2007).

Study population	Males				Females				Total		Percent of full roster
	Whites		Nonwhites		Whites		Nonwhites				
	Cases	Employees (%)	Cases	Employees (%)	Cases	Employees (%)	Cases	Employees (%)	Cases	Employees (%)	
Full roster	7	2723 (72.3%)	0	570 (15.1%)	0	358 (9.5%)	0	117 (3.1%)	7	3768 (100%)	100.00
Examinations	7	1885 (69.5%)	0	467 (17.2%)	0	271 (10.0%)	0	89 (3.3%)	7	2712 (100%)	71.97
Examinations and work histories	7	1771 (69.5%)	0	441 (17.3%)	0	253 (9.9%)	0	84 (3.3%)	7	2549 (100%)	67.65
Criminal investigators with examinations and work histories	7	1715 (69.2%)	0	436 (17.6%)	0	244 (9.8%)	0	83 (3.4%)	7	2478 (100%)	65.76

**Table 2:** Distribution of self-reported bladder cancer cases and of person-years observed for white males in full roster cohort by calendar period and age group.

Age group	1993–1997		1998–2002		2003–2007		Total	
	Cases	Person-years	Cases	Person-years	Cases	Person-years	Cases	Person-years
0–4	0	0.0	0	0.0	0	0.0	0	0.0
5–9	0	0.0	0	0.0	0	0.0	0	0.0
10–14	0	0.0	0	0.0	0	0.0	0	0.0
15–19	0	0.0	0	0.0	0	0.0	0	0.0
20–24	0	25.1	0	81.6	0	71.3	0	178.0
25–29	0	642.9	0	611.6	0	749.7	0	2004.1
30–34	2	1730.8	0	1593.4	0	1670.6	2	4994.8
35–39	0	1321.5	0	2136.4	1	2125.1	1	5583.0
40–44	0	773.1	2	1329.7	0	2111.9	2	4214.6
45–49	0	1574.9	1	763.0	0	1291.9	1	3629.8
50–54	0	1046.2	1	1083.5	0	571.5	1	2701.1
55–59	0	261.6	0	383.1	0	264.5	0	909.2
60–64	0	12.4	0	113.1	0	95.9	0	221.4
65–69	0	3.9	0	5.7	0	43.3	0	52.9
70–74	0	1.8	0	2.7	0	3.0	0	7.5
75–79	0	0.0	0	0.0	0	0.0	0	0.0
80–84	0	0.0	0	0.0	0	0.0	0	0.0
85–89	0	0.0	0	0.0	0	0.0	0	0.0

Total	2	7394.0	4	8103.8	1	8998.7	7	24496.5

*The one remaining unverified cancer case occurred in the 1998–2002 time frame.

**Table 3:** Standardized incidence ratios (SIRs) of self-reported and verified urinary bladder cancer cases for the period 1993–2007.

Study population	Employee #	Person-years	Expected cases*	Observed cases	SIR	95% CI	
Entire roster cohort	3,768	34,818.01	2.91	7	2.41	1.17	4.96
Entire roster cohort**	3,768	34,818.01	2.91	5	1.72	0.73	4.02
Roster white males	2,723	24,496.47	2.39	7	2.93	1.42	6.07
Roster white males**	2,723	24,496.47	2.39	5	2.09	0.90	1.91
Exam white males	1,885	19,648.25	1.15	7	6.08	2.94	12.54
Exam white males**	1,885	19,648.25	1.15	5	4.34	1.85	10.16
Job 1811 white males	1,715	17,976.42	0.92	7	7.63	3.70	15.75
Job 1811 white males**	1,715	17,976.42	0.92	5	5.45	2.33	12.76

*Expected number of cases calculated using US incidence rates from SEER for the same period.

**SIRs determined for the five verified cases with pathology reports.

**Table 4:** Age-specific white male bladder cancer incidence rates (1/100,000) for ATF and SEER (13 registries), with respective rate ratios, for the period 1993–2007.

Age	ATF	SEER	RR	95% CI-L	95% CI-U
20–24	0.00	0.37	0.00	0.00	0.00
25–29	0.00	0.57	0.00	0.00	0.00
30–34	40.04	1.17	34.32	3.78	124.24
35–39	17.91	2.80	6.40	0.19	35.63
40–44	47.45	5.83	8.13	0.89	29.45
45–49	27.55	13.13	2.10	0.06	11.68
50–54	37.02	25.33	1.46	0.04	8.14
55–59	0.00	50.57	0.00	0.00	0.00
60–64	0.00	88.33	0.00	0.00	0.00
65–69	0.00	145.70	0.00	0.00	0.00
70–74	0.00	209.43	0.00	0.00	0.00
75–79	0.00	275.40	0.00	0.00	0.00
80–84	0.00	327.83	0.00	0.00	0.00
85–89	0.00	353.73	0.00	0.00	0.00
